# Draft Whole-Genome Sequences of 11 *Bacillus cereus* Food Isolates

**DOI:** 10.1128/genomeA.00485-16

**Published:** 2016-06-02

**Authors:** Hasmik Hayrapetyan, Jos Boekhorst, Anne de Jong, Oscar P. Kuipers, Masja N. Nierop Groot, Tjakko Abee

**Affiliations:** a Laboratory of Food Microbiology, Wageningen University, Wageningen, The Netherlands; b Molecular Genetics, University of Groningen, Groningen, The Netherlands; c NIZO food research, Ede, The Netherlands; d Wageningen UR Food and Biobased Research, Wageningen, The Netherlands; e Top Institute Food and Nutrition (TIFN), Wageningen, The Netherlands

## Abstract

*Bacillus cereus* is a foodborne pathogen causing emetic and diarrheal-type syndromes. Here, we report the whole-genome sequences of 11 *B. cereus* food isolates.

## GENOME ANNOUNCEMENT

*Bacillus cereus* is a spore-forming foodborne pathogen that is ubiquitously present in the environment, showing high capacity to adapt to different environmental niches (1, 2). *B. cereus* is closely related to *Bacillus anthracis*, the causative agent of anthrax, and to the insect pathogen *Bacillus thuringiensis* (3). Soil is the main reservoir of *B. cereus* spores, and food can serve as a vehicle to transfer them to the host (4). *B. cereus* is the causative agent of two types of toxin-associated foodborne diseases: emetic and diarrheal syndromes (5). The emetic syndrome is an intoxication caused by the thermostable emetic toxin cereulide (6). The emetic toxin is produced by vegetative *B. cereus* cells in food before ingestion and remains active upon stomach transit. It is toxic to mitochondria by acting as a potassium ionophore and has been reported to inhibit human natural killer cells. The diarrheal syndrome is caused by enterotoxins secreted by vegetative cells in the small intestine, where they can act by disrupting the integrity of the membrane of epithelial cells (7).

Eleven *B. cereus* strains isolated from different food sources (8) were grown overnight (18 h) with shaking (200 rpm) in 10 ml of brain heart infusion (BHI) broth (Becton, Dickinson) at 30°C. Two milliliters of this culture were centrifuged at 13,000 × *g* to harvest the cells, and the resulting cell pellet was resuspended in 50 mM EDTA (pH 8.0). Genomic DNA of the strains was isolated using the Wizard genomic DNA purification kit (Promega, Madison, WI), according to the manufacturer’s instructions.

The isolated DNA was sheared to 250- to 350-bp fragments and paired-end sequenced on an Illumina HiSeq 2000 outsourced to BaseClear (Leiden, The Netherlands). CLC Genomics Workbench version 6.0.1 (CLC bio), SSPACE version 2.3 (PMID 21149342), and GapFiller version 1.1 (PMID 23095524) were used for assembly. The RAST server (9) (PMID 18261238) was used to annotate the genomes.

### Nucleotide sequence accession numbers.

The genome sequences of the 11 *B. cereus* strains have been deposited at DDBJ/EMBL/GenBank under the accession numbers listed in Table 1. The version described in this paper is the first version.

**TABLE 1:** Sequenced *B. cereus* strains and their isolation sources

*B. cereus* strain^a^	Isolation source	Accession no.
B4079	Canned chocolate beverage	LJIT00000000
B4081	Provolone sauce	LJJZ00000000
B4082	Asparagus ham sauce	LJKA00000000
B4083	Tortellini con fungi	LJKB00000000
B4084	Indian rice dish	LJKC00000000
B4085	Asparagus soup	LJKD00000000
B4088	Dressing	LJKE00000000
B4116	White sauce	LJKF00000000
B4118	Ice cream	LJKH00000000
B4120	Water	LJKI00000000
B4155	Beef salad	LJKJ00000000

a B-numbers refer to the strain collection at NIZO food research.

## References

[1] Abee T, Nierop Groot M, Tempelaars M, Zwietering M, Moezelaar R, van der Voort M 2011 Germination and outgrowth of spores of *Bacillus cereus* group members: diversity and role of germinant receptors. Food Microbiol 28:199–208. doi:10.1016/j.fm.2010.03.015. 21315974

[2] Guinebretière MH, Thompson FL, Sorokin A, Normand P, Dawyndt P, Ehling-Schulz M, Svensson B, Sanchis V, Nguyen-The C, Heyndrickx M, De Vos P 2008 Ecological diversification in the *Bacillus cereus* group. Environ Microbiol 10:851–865. doi:10.1111/j.1462-2920.2007.01495.x. 18036180

[3] Rasko DA, Altherr MR, Han CS, Ravel J 2005 Genomics of the *Bacillus cereus* group of organisms. FEMS Microbiol Rev 29:303–329. 15808746 10.1016/j.femsre.2004.12.005

[4] Ceuppens S, Boon N, Uyttendaele M 2013 Diversity of *Bacillus cereus* group strains is reflected in their broad range of pathogenicity and diverse ecological lifestyles. FEMS Microbiol Ecol 84:433–450. doi:10.1111/1574-6941.12110. 23488744

[5] Stenfors Arnesen LP, Fagerlund A, Granum PE 2008 From soil to gut: *Bacillus cereus* and its food poisoning toxins. FEMS Microbiol Rev 32:579–606. doi:10.1111/j.1574-6976.2008.00112.x. 18422617

[6] Ehling-Schulz M, Frenzel E, Gohar M 2015 Food-bacteria interplay: pathometabolism of emetic *Bacillus cereus*. Front Microbiol 6:704. doi:10.3389/fmicb.2015.00704. 26236290 PMC4500953

[7] Jeßberger N, Krey VM, Rademacher C, Böhm ME, Mohr AK, Ehling-Schulz M, Scherer S, Märtlbauer E 2015 From genome to toxicity: a combinatory approach highlights the complexity of enterotoxin production in *Bacillus cereus*. Front Microbiol 6:560. doi:10.3389/fmicb.2015.00560. 26113843 PMC4462024

[8] Hayrapetyan H, Muller L, Tempelaars M, Abee T, Nierop Groot M 2015 Comparative analysis of biofilm formation by *Bacillus cereus* reference strains and undomesticated food isolates and the effect of free iron. Int J Food Microbiol 200:72–79. doi:10.1016/j.ijfoodmicro.2015.02.005. 25700364

[9] Aziz RK, Bartels D, Best AA, DeJongh M, Disz T, Edwards RA, Formsma K, Gerdes S, Glass EM, Kubal M, Meyer F, Olsen GJ, Olson R, Osterman AL, Overbeek RA, McNeil LK, Paarmann D, Paczian T, Parrello B, Pusch GD, Reich C, Stevens R, Vassieva O, Vonstein V, Wilke A, Zagnitko O 2008 The RAST server: Rapid Annotations using Subsystems Technology. BMC Genomics 9:75. doi:10.1186/1471-2164-9-75. 18261238 PMC2265698

